# Metacognition and confidence: comparing math to other academic subjects

**DOI:** 10.3389/fpsyg.2015.00742

**Published:** 2015-06-02

**Authors:** Shanna Erickson, Evan Heit

**Affiliations:** Department of Cognitive and Information Sciences, University of California, MercedMerced, CA, USA

**Keywords:** metacognition, math anxiety, math education, confidence

## Abstract

Two studies addressed student metacognition in math, measuring confidence accuracy about math performance. Underconfidence would be expected in light of pervasive math anxiety. However, one might alternatively expect overconfidence based on previous results showing overconfidence in other subject domains. Metacognitive judgments and performance were assessed for biology, literature, and mathematics tests. In Study 1, high school students took three different tests and provided estimates of their performance both before and after taking each test. In Study 2, undergraduates similarly took three shortened SAT II Subject Tests. Students were overconfident in predicting math performance, indeed showing greater overconfidence compared to other academic subjects. It appears that both overconfidence and anxiety can adversely affect metacognitive ability and can lead to math avoidance. The results have implications for educational practice and other environments that require extensive use of math.

## Introduction

Two components of metacognition are particularly relevant for successful learning: self-monitoring, e.g., assessing performance, and self-regulation, e.g., choosing what and how to study (Nelson and Dunlosky, 1991; Thiede et al., 2003; Metcalfe, 2009). Metacognition has been found to be crucial for calibration of self-knowledge of ability (Schunk and Ertmer, 2000; Sperling et al., 2004), in domains and tasks such as mathematics (Pugalee, 2001), science (Schraw et al., 2006), reading (Pressley, 2002), and writing (Pugalee, 2001). If people are unable to assess their performance accurately, then it is unlikely that they will be able to learn optimally (see Townsend and Heit, 2011, for a related argument). Any improvements in metacognition would allow learners to better judge what they know and how well they will be able to learn information and recall it later.

Our focus in the present research is self-monitoring in math. In addressing the topic of metacognition and math, it is important to consider whether metacognition is domain-general or domain-specific. In other words, to what extent are there general points to be made about people’s metacognitive abilities, potential for error, and underlying mechanisms across subject domains, and to what extent are there distinctive points to be made for particular domains? Do the difficulties that learners face with math reflect general issues with metacognition, or something special about math?

Though there is ongoing debate on whether metacognition is domain-specific or domain-general, there is no debate on the importance of metacognition in any learning process. In this paper we will focus primarily on possible differences between metacognition in mathematics learning when compared to other domains, as math anxiety can affect both domain performance and metacognitive performance.

Based on past research, it remains unclear whether metacognitive performance is similar across domains. Metacognition research typically addresses a single subject domain in isolation, and those few studies comparing metacognition across domains have shown mixed results (e.g., Veenman et al., 2006). Though these past studies have used varying methodologies and assessments for measuring metacognition, results still bear on this issue of domain generality versus specificity and provide two views which we must consider.

### Domain-General Views of Metacognition

Some studies support domain generality of metacognition, treating it as a skill that can be applied across different content areas (e.g., Schraw, 1996; Halpern, 1998; Veenman and Verheij, 2001). In these cases, domain-general metacognitive skills are distinguished from domain-specific knowledge. This framework assumes that cognitive skills can be domain-specific whereas metacognitive skills can be applied across even unrelated domains. Interestingly, metacognitive ability appears unrelated to IQ (Alexander et al., 1995). Rather, metacognitive skills are assumed to improve along with domain knowledge.

Metacognitive skills are further related to domain knowledge in that metacognitive skill can aid learners those with low ability or knowledge. For instance, Swanson (1990) showed that metacognitive ability compensated for IQ in a comparison between fifth and sixth grade student problem solving ability. Ability to solve problems was unrelated to IQ, while those with higher metacognitive ability were better able to solve problems than those with lower metacognitive ability. This result suggests that metacognition can be applied flexibly across tasks and thus is a domain-general skill.

This domain-general view of metacognition is consistent with the unskilled and unaware phenomenon, in which people show domain-general overconfidence in their abilities, with low performers showing greater overconfidence than higher performers (Kruger and Dunning, 1999; Dunning et al., 2003). This phenomenon has been shown for students predicting performance on laboratory tests ranging from logical reasoning to grammatical knowledge and sense of humor. Studies with academic content in classroom settings also exhibit this phenomenon (Maki and Berry, 1984; Miller and Geraci, 2011). Miller and Geraci (2011) also demonstrated that the lowest performers were overconfident in exam score predictions, but they were also less confident in these predictions than were the highest performers. Thus, although the unskilled might be more aware than once thought, they still demonstrate overconfidence nonetheless. Furthermore, while people with high performance might demonstrate slight underconfidence, people with lower performance have even more exaggerated overconfidence. From this, one might predict that students who are struggling in math are particularly overconfident.

### Domain-Specific Views of Metacognition

In contrast, some investigations of individual differences in metacognition point toward domain specificity. For example, Kelemen et al. (2000) found that metamemory accuracy was task specific for university students. They tested memory monitoring performance across four metacognitive tasks: ease of learning judgments for Swahili-English word pairs, feeling of knowing judgments for general knowledge questions, judgments of learning for unrelated English word pairs, and text comprehension monitoring for narrative texts. While they found individual differences in memory and confidence that remained constant across tasks, individual differences in metacognitive accuracy changed for each task. Glaser et al. (1992) provided evidence that metacognition can differ based on task. They found that metacognitive strategies of university students varied across discovery learning tasks. In their comparison of several reasoning tasks, they further found variability in metacognitive performance for components of problem solving. There are a variety of alternative but equally successful problem solving strategies and a variety of metacognitive approaches for these problems. In general, successful problem solvers use metacognitive strategies more often than less successful problem solvers, but there is no one set of metacognitive strategies that led to successful problem solving. They also found that domain content and context led to variation in use of metacognitive strategies, and that particular metacognitive skills were associated with specific learning success or failure within particular domains and contexts.

Still others have suggested that metacognition might be domain-specific early in development, beginning as reflective self-analysis of cognition. For example, Paris and Byrnes (1989) suggest that such self-directed reflection develops in children as self-corrections, and this behavior becomes more prominent as children get older. As children develop self-regulation for individual tasks, then gradually learn to apply general self-correction skills across a variety of tasks. Similarly, Karmiloff-Smith (1992) suggested that this reflection results in the restructuring of self-knowledge that increases theoretical understanding of one’s cognition. This restructuring starts to differentiate and separate various domains of knowledge. Both these views support a theory of first domain-specificity of metacognition that eventually extends to be a domain-general skill. Then as metacognitive skill improves, this skill can be applied across a variety of domains. Veenman and Spaans (2005) provided evidence for this in their findings of first domain-specificity in metacognitive skill in the first year of high school then domain generality later for third year high school students. First-year and third-year students solved math problems while thinking aloud and also performed an inductive learning biology task. Metacognitive skillfulness was measured based on enactment of metacognitive behaviors (e.g., entirely reading a problem statement, selection of relevant information needed to solve the problem, monitoring the on-going problem-solving process, checking the answer, reflecting on the answer). A difference in metacognitive skillfulness was observed when comparing the two groups of students in that metacognitive skills are at first domain-specific for first-year students, while they are domain-general for third-year students.

Previous research shows connections between metacognition and math anxiety. Math anxiety (or math phobia) is a fear of math that leads to math avoidance or lower math performance (Ashcraft, 2002; Ashcraft and Krause, 2007). This sometimes extreme anxiety is harmful in both educational and workplace settings (Meece et al., 1990; Furner and Berman, 2003). Performing math tasks in stressful situations, such as during tests, only compounds math anxiety (Beilock and Carr, 2005; Beilock, 2008). This anxiety can start early in children’s education, with elementary school students already showing harmful effects of math anxiety on their math achievement (Ramirez et al., 2013). Math anxiety interrupts cognitive processing through its interference with working memory, and this is what can cause people to show lower performance under pressure (Beilock and Carr, 2005). While math anxiety does not appear to affect simple math tasks such as single digit addition, it does affect decision-making processes for number sense and any task that required procedural aspects of arithmetic (Dehaene, 1997; Ashcraft, 2002). The tasks requiring use of working memory are adversely affected by math anxiety. Students who are highly math anxious tend to make more errors in timed problems than did those with low math anxiety. This is also consistent with Eysenck and Calvo’s (1992) model of general anxiety effects, in which general anxiety disrupts working memory through preoccupation with thoughts and attention given to worry instead of to the current task. This preoccupation is a second task that places a heavier load on working memory, a component of cognition that is used in metacognition (Shimamura, 2000).

The widespread evidence for math anxiety suggests the view that math may be uniquely problematic compared to other academic subjects. There does not seem to be a corresponding body of evidence for, say, literature anxiety or even biology anxiety. Generalizing this point, we would expect that if students fear math, then they should generally have low confidence in math compared to other subjects and a corresponding difference in metacognitive ability. Indeed, Ashcraft (2002) found evidence for this point in terms of strong negative correlations between math anxiety and self-confidence in math.

### Overview of Experiments

Accounts of domain specificity are consistent with the idea that math is uniquely problematic. Contrasting this view that math is unique are accounts of domain generality, including the unskilled and unaware phenomenon. So are students underconfident in math, as would be expected from the math is unique view, or are students overconfident in math, as would be predicted by the unskilled and unaware view? Note that we present these as opposing views, but the predictions from these views were derived by ourselves.

Our purpose is not to examine whether math-phobic students are less confident than non-math-phobic students or whether students are less confident in math than in other subjects. Rather, we focus on calibration of metacognitive judgments, that is, whether students are under- or over-confident relative to their performance. In addition to general measures of calibration, we also compared calibration across academic subjects. We assessed both absolute calibration, in which we simply measure how well subjective scores matched objective scores, as well as relative calibration, assessing whether participants with higher subjective scores had correspondingly higher objective scores. Other than looking at confidence for students with lower versus higher scores, it was not our aim to examine individual differences.

In two studies, we assessed confidence in math as well as other academic subjects (biology, literature). These studies were conducted based on approval by the Institutional Review Board (IRB) for our institution (University of California, Merced). High school (Study 1) and college (Study 2) students took standardized tests and estimated their performance. Following the view that math is unique, we would expect underconfidence in math relative to the other subjects and likely worse metacognitive calibration. In contrast, following the unskilled and unaware view, we would expect similar overconfidence in math compared to other subjects. In Study 2, we included a standard measure of math anxiety. We note again that it was not our purpose to examine individual differences in anxiety but rather to look at students who were most likely math anxious overall, and in general our findings are limited to the groups we studied.

## Study 1

### Method

Participants in this study attended a summer program at a diverse public high school in California. They made two estimates: predictions (before each test) of their performance as well as postdictions (after each test). Mutiple choice tests were adapted from teachers’ materials used at that grade level in this school, giving students a basis for making predictions, with even more information when making postdictions.

Our main focus was to compare calibration of estimates about math to the other two subjects. We also compared predictions to postdictions, allowing us to determine if metacognitive judgments improved after completing a test, as would be expected from previous research (Kruger and Dunning, 1999; Dunning et al., 2003).

#### Participants

There were 40 participants (25 female, 15 male). All were students (*mean age* = 15.27, SD = 0.55) at Central Valley High School in Ceres, California, who took the study for extra credit in their summer school class (Algebra 1). A majority of these students had failed math the previous academic year. Ceres Unified School District is located in a rural area; its student population is 72% Hispanic-Latino, 21% White-Caucasian, 3% Asian-Pacific Islander, 1% African American, and 3% of other ethnicity.

#### Materials and Procedure

Each participant took three computer-based tests (biology, literature, mathematics, in randomized order), each test consisting of 15 questions with 15 min allowed per test. Questions were normed so that the overall level of difficulty across tests was comparable, to avoid ceiling and floor effects and to assure variance in scores. Questions left unfinished within the allotted time were scored as incorrect. Participants were told that these tests were similar to those from their current classes. Question style was multiple choice with five answer choices. Before each test, participants provided a predicted score (number of questions correct) for how well they would do. After each test, participants provided a postdicted score for how well they thought they had performed. They were not told their actual scores.

### Results and Discussion

**FIGURE 1:**
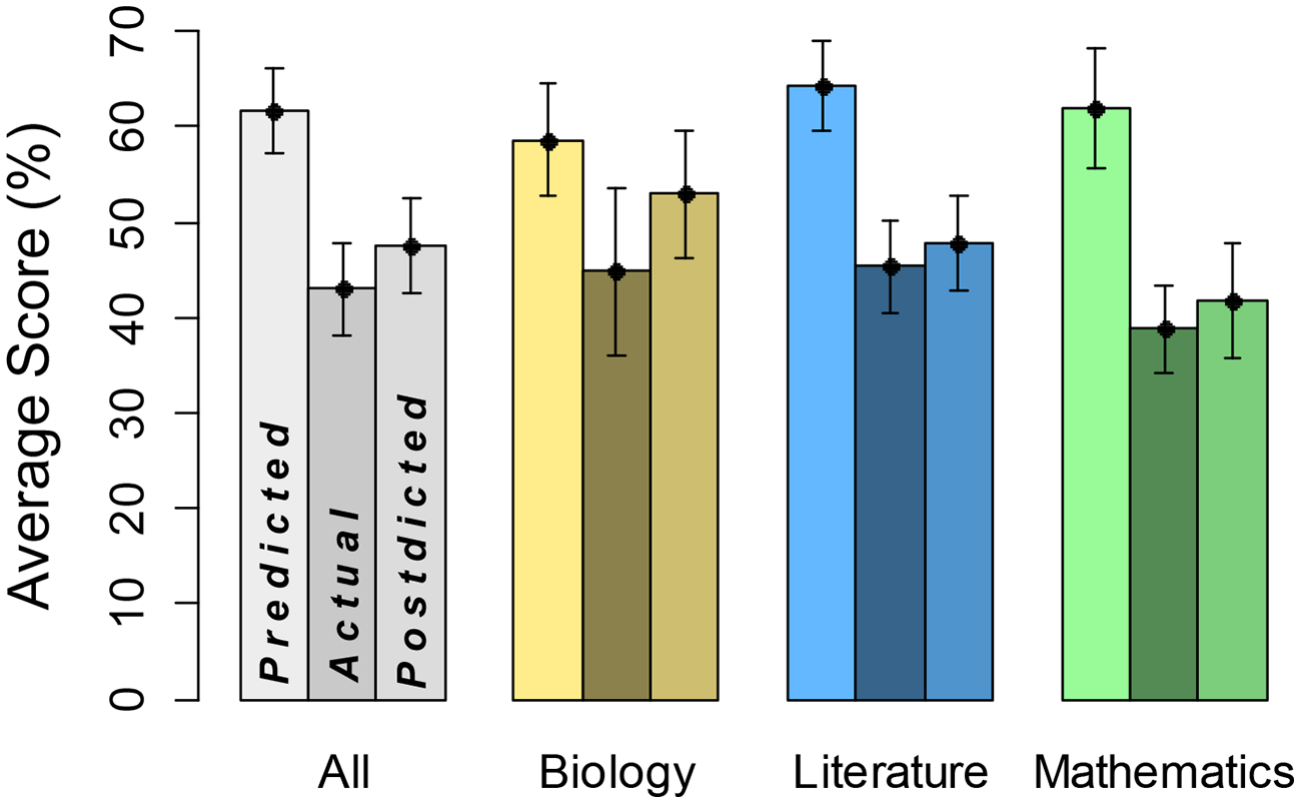
**Predicted, actual, and postdicted scores by domain**. Leftmost bars represent predicted scores, middle bars domain performance, and rightmost bars postdicted scores, each with SE.

Key descriptive results are in **Figure 1**. The leftmost bar for each category represents average predicted score, the middle bar represents average actual test score, and the rightmost bar represents average postdicted score. The general pattern is that predictions are substantially greater than actually performance, and postdictions are somewhat greater than actually performance. This overconfidence is particularly striking for predictions about math performance. We do not show breakdown by gender, however, predicted, actual, and postdicted scores averaged about 10% higher for males.

First, we examined predictions in a three-way, predicted versus actual score × academic subject × gender, ANOVA. There was a significant main effect of predicted (*mean* = 61.7) versus actual (*mean* = 43.0) score, *F*(1,38) = 54.45, *MSE* = 6.00, η^2^ = 0.59, *p* < 0.0001, indicating overconfidence in predictions. The academic subject variable did not reach statistical significance, *F*(2,152) = 4.14, *MSE* = 4.11, η^2^ = 0.05. However, there was a significant predicted versus actual score × academic subject interaction, *F*(2,152) = 10.31, *MSE* = 4.11, η^2^ = 0.11, *p* < 0.0001, implying that overconfidence differed by academic subject. Notably, participants showed the highest degree of overconfidence in mathematics (*predicted score* = 62.0, *actual score* = 38.9). There was also a main effect of gender, *F*(1,38) = 5.88, *MSE* = 17.41, η^2^ = 0.13, *p* < 0.05, but remaining interaction terms were not statistically significant, *F* < 1. Hence, degree of overconfidence did not depend on gender, although it may be that the sample size did not yield enough power to fully address this point.

We also conducted a comparable analysis on postdicted scores (*mean* = 47.5). In this three-way ANOVA, there was a significant effect of academic subject, *F*(2,152) = 21.00, *MSE* = 4.06, η^2^ = 0.21, *p* < 0.0001. Note that biology had the highest values overall and mathematics had the lowest. There was also a significant main effect of gender, *F*(1,38) = 5.10, *MSE* = 19.74, η^2^ = 0.12, *p* < 0.05. The remaining main effect and interaction terms were not statistically significant, *F* < 1. Hence, we did not see significant overall overconfidence on postdictions, and overconfidence did not depend on academic subject or gender.

The preceding analyses focused on absolute calibration, namely how well subjective scores matched objective scores, on average. We next examined relative calibration, namely whether participants with higher subjective scores had correspondingly higher objective scores, measuring relative calibration in terms of correlation coefficient, *r*, across all participants (**Table 1**). Relative calibration is particularly strong for math, and particularly weak for literature, with biology falling between.

**Table 1:** Correlations between participant-produced estimates and performance by domain.

	Biology	Literature	Math
Prediction versus actual score	0.32^*^	0.10	0.56^***^
Postdiction versus actual score	0.39^*^	0.21	0.64^***^

^*^p <0.01; ^**^p <0.05; ^***^p <0.001 (as compared to 0).

Correlations of estimates of performance were not significant across domains, suggesting that student metacognition was not domain-general. We also tested differences in correlations both across domain and within domain. Steiger *z*-tests of independent correlations showed that the prediction versus actual score correlations are significantly different between math and literature (*z* = 2.28, *p* < 0.02). Similarly, postdiction versus actual score correlations are significantly different between math and literature (*z* = 2.36, *p* < 0.02). Williams *t*-tests of dependent correlations reveal that differences between prediction versus actual score and postdiction versus actual score are significant for all of biology (*t* = -3.19, *p* < 0.0029), literature (*t* = -3.13, *p* < 0.0034), and math (*t* = -2.28, *p* < 0.028). Thus calibration significantly improved in postdictions on all tests when compared to calibration of predictions.

**FIGURE 2:**
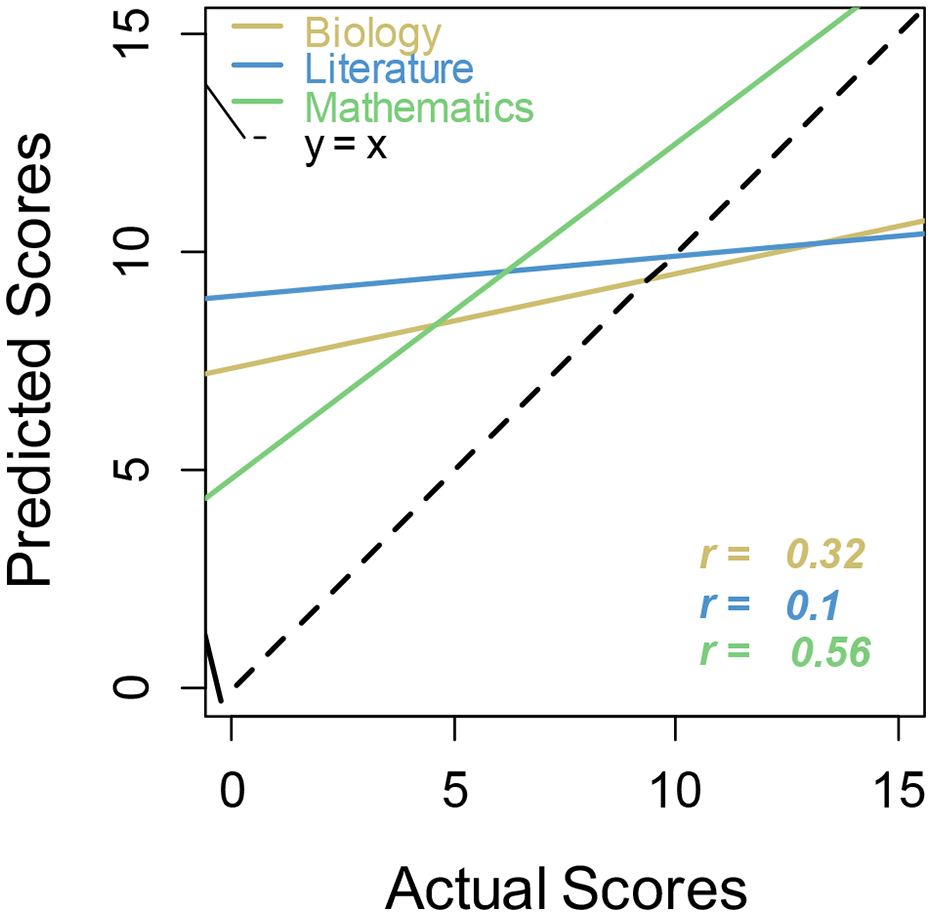
**Regression lines for actual versus predicted scores by academic subject**. Regression lines that more closely follow the main dashed line (perfect calibration) indicate better metacognitive calibration for that academic subject.

Linear regression calibration curves also illustrate this point (**Figure 2**). Regression lines that more closely follow the main dashed line (perfect calibration) indicate better metacognitive calibration for that academic subject. Regression lines for biology and literature are shallow, showing little sensitivity to actual performance. These lines show the usual unskilled and unaware pattern of overconfidence at the lowest level of performance and underconfidence at the highest level (Kruger and Dunning, 1999; Dunning et al., 2003). Calibration for math in this case seems to take unskilled and unaware one step further, in that even the high performers over-estimated their ability.

In general, results more closely supported the unskilled and unaware view rather than the math is unique view. Students were generally overconfident in all three academic subjects, at least on predictions if not postdictions. The only evidence we found suggesting a difference for math is that relative calibration for math was actually the best and overconfidence was the greatest. Overall performance was somewhat lower for female students than for males, but so were predictions and postdictions, so their overconfidence was no different.

## Study 2

We turn to another study, attempting to replicate and extend key findings from Study 1, which might have been due to idiosyncrasies of the particular student sample or test instruments used. In Study 2, we conducted a similar study on college students, using sample SAT test questions. We would have expected high school students in Study 1 to be math anxious, because most high school students show some math anxiety (Hembree, 1990; Ma, 1999; Maloney and Beilock, 2012), and most of the students in our study had previously failed math classes. In Study 2, we included a standard measure of math anxiety adapted from a shortened Math Anxiety Ratings Scale (MARS, Alexander and Martray, 1989). Although we did not directly measure math anxiety for students in Study 1, we replicated this study within the same school population the following summer, and the average shortened MARS score was 77. This study also included additional measures but otherwise replicated findings from the high school study presented here. For comparison, Ashcraft and Moore (2009) found an average shortened MARS score of 61 across several college samples identified as math anxious.

### Method

#### Participants

There were 46 participants (28 female, 18 male) in this study. All were UC Merced undergraduates (*mean age* = 19.96, SD = 1.75) who received extra credit in their introductory psychology or cognitive science classes for their participation. The UC Merced undergraduate population is 40% Hispanic-Latino, 29% Asian-Pacific Islander, 17% White-Caucasian, 7% African American, and 7% other ethnicity.

#### Materials and Procedure

Participants took three tests (biology, literature, mathematics), each with 15 questions. Again, questions left unfinished within the allotted time were scored as incorrect. Participants were told that these were based on SAT II Subject Tests (which most students have taken). Students completed three assessments derived from past questions released by the College Board, making predictions and postdictions as in Study 1. Test questions were normed to avoid ceiling and floor effects. Then, participants answered 23 questions about math anxiety from a variant of the shortened MARS (Alexander and Martray, 1989), using a rating scale ranging from 1 – “no anxiety” to 5 – “very high anxiety,” with possible scores from 23 to 115. (We dropped two of three questions from the standardized shortened MARS with nearly identical wording).

In pilot studies, in an effort to design tests of equal difficulty, items of comparable difficulty for each test were selected using individual question ratings (easy, medium, difficult) provided by College Board. However, performance floor effects on the pilot math tests were so pronounced that easier questions were substituted in an effort to bring average performance closer to the level as the literature and biology tests.

### Results and Discussion

**FIGURE 3:**
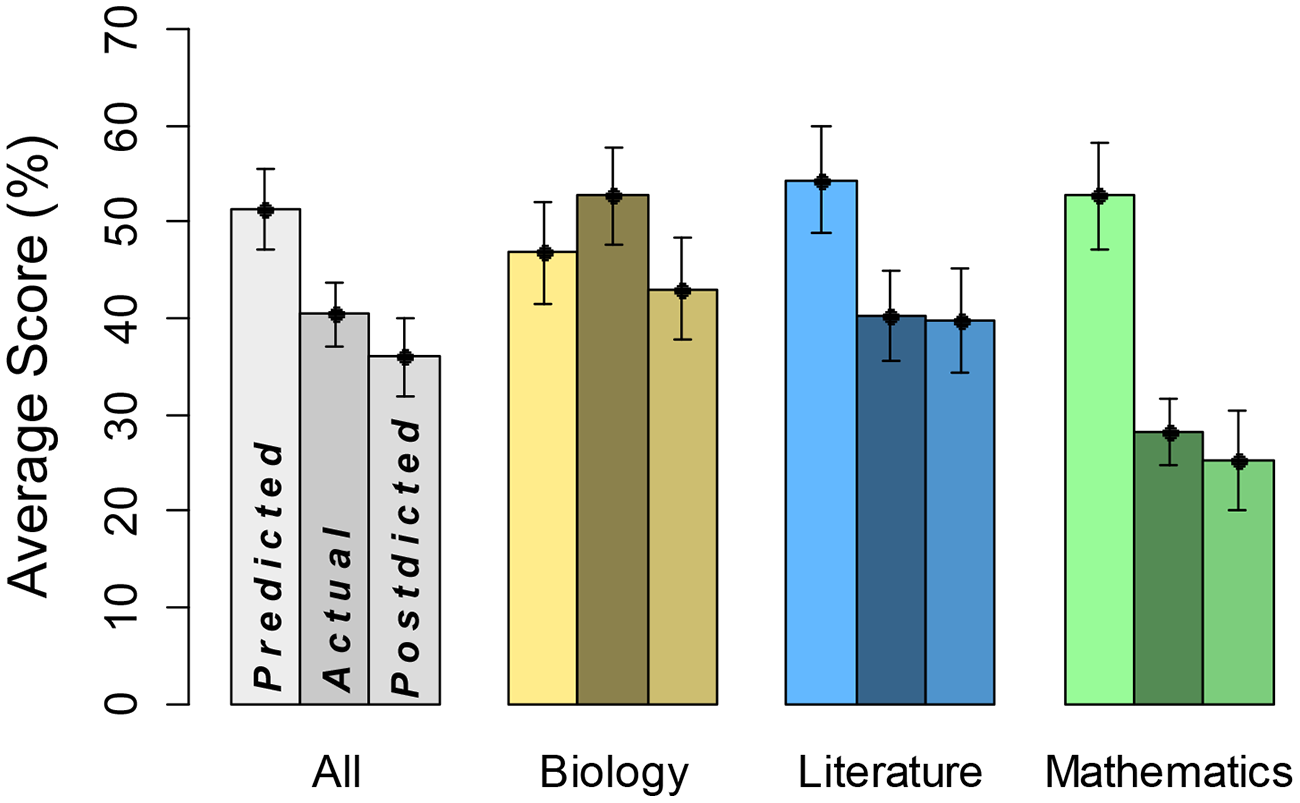
**Predicted, actual, and postdicted scores by domain**. Leftmost bars represent predicted scores, middle bars domain performance, and rightmost bars postdicted scores, each with SE.

The results largely replicated Study 1 (see **Figure 3**), most notably in terms of general over-confidence of predictions compared to actual scores, most notably for math.

In a three-way, predicted versus actual score × academic subject × gender ANOVA, there was a significant main effect of predicted (*mean* = 51.3) versus actual (*mean* = 40.4) score, *F*(1,44) = 19.70, *MSE* = 9.31, η^2^ = 0.31, *p* < 0.0001, indicating overconfidence in predictions. There was also a significant main effect of academic subject, *F*(2,176) = 7.19, *MSE* = 4.18, η^2^ = 0.09, *p* < 0.0001; scores were lowest overall in math. There was a significant interaction between these two variables, *F*(2,176) = 29.58, *MSE* = 4.18, η^2^ = 0.22, *p* < 0.0001, indicating that degree of overconfidence depended on academic subject. Overconfidence was greatest in mathematics (*predicted score* = 52.8, *actual score* = 28.3), however, we are careful not to over-interpret the interaction because actual scores also differed by academic subject. The remaining main effect (gender) and interaction terms were not statistically significant, *F* < 1. Hence, the finding of overconfidence, particularly in math, did not depend on gender.

We also conducted a comparable analysis on postdicted scores (*mean* = 36.0) and actual scores. This ANOVA revealed a main effect of postdicted versus actual score, *F*(1,44) = 4.48, *MSE* = 6.85, η^2^ = 0.09, *p* < 0.05, indicating that participants were slightly yet significantly underconfident overall. With that said, given the overall drop from predictions to postdictions, we see this as reflecting that participants were better calibrated after taking the test, as in Study 1. (A third ANOVA comparing predicted and postdicted scores showed a main effect of predicted versus postdicted.) There was a main effect of academic subject, *F*(2,176) = 37.52, *MSE* = 3.70, η^2^ = 0.41, *p* < 0.0001. There was also a significant interaction between academic subject and postdicted versus actual score, *F*(2,176) = 3.12, *MSE* = 3.70, η^2^ = 0.02, *p* < 0.05, indicating a difference in overconfidence by academic domain. Though all medium and difficult questions were removed and replaced with easy ones (based on College Board question ratings), performance remained lower for the math test compared to the other two subjects. The remaining main effect (gender) and interaction terms were not statistically significant, *F* < 4. Even after replacing all questions with those rated as easy by the College Board, and thus creating a minimally difficult assessment that incorporated actual SAT questions, math test scores were consistently lower than biology or literature test scores. Analyses were also conducted using a sample of these math questions by selecting seven questions that participants scored best on. However, this did not change our findings. Again as in Study 1, the finding of overconfidence, particularly in math, did not depend on gender.

**FIGURE 4:**
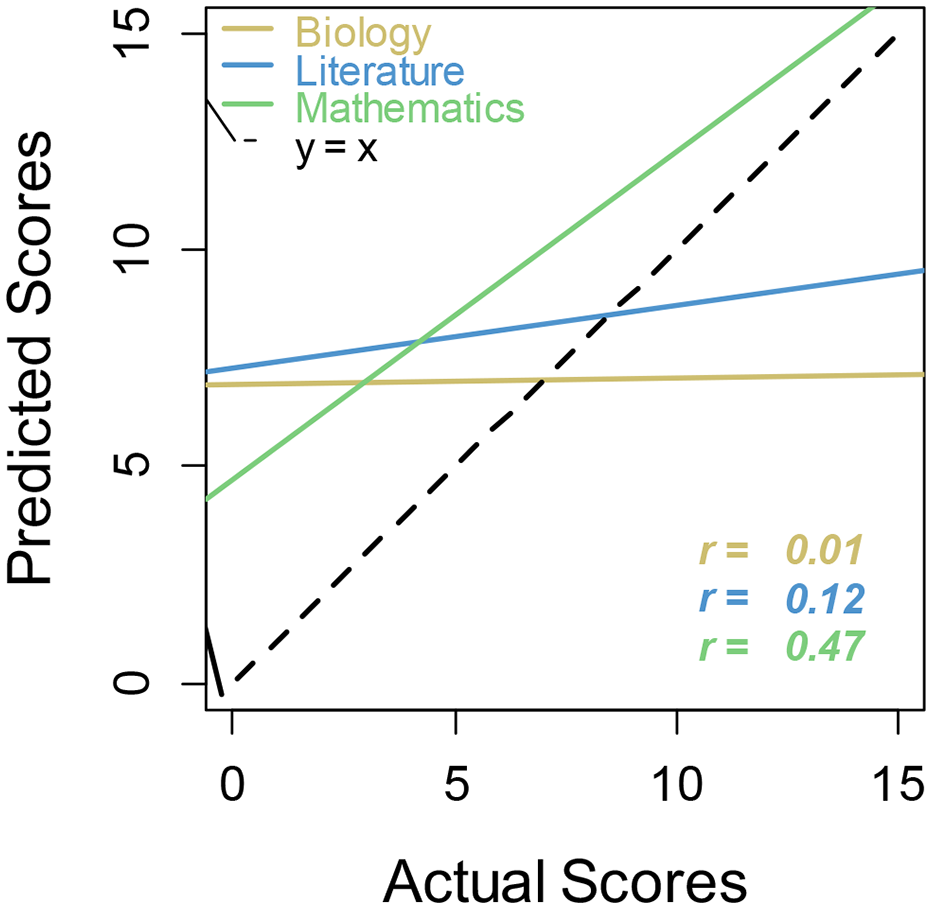
**Regression lines for actual versus predicted scores by academic subject**. Regression lines that more closely follow the main dashed line (perfect calibration) indicate better metacognitive calibration for that academic subject.

Analyses of relative calibration (**Table 2**, **Figure 4**) yielded results similar to Study 1. Correlations between predicted and actual scores were highest for math, although, based on a Steiger *z*-test of independent correlations, there was only a significant difference in correlations between predicted and actual score for biology and predicted versus actual score for math (*z* = 2.32, *p* < 0.02). Correlations for postdicted scores were approximately the same for all three academic subjects, and there were no significant differences among these correlations. Williams *t*-tests of dependent correlations reveal that there were significant differences in predicted versus actual and postdicted versus actual correlations for both literature (*t* = -3.21, *p* < 0.0025) and biology (*t* = –3.82, *p* < 0.00042). Thus calibration was significantly different before and after the test for both literature and biology. However, this pair of correlations for math showed no significant difference, hence there was a lack of evidence for improved calibration in math. Again, the regression lines for biology and literature showed overconfidence at the lowest level of performance and underconfidence at the highest level, revealing again the general unskilled and unaware pattern. In contrast, students were simply overconfident in general for math.

**Table 2:** Correlations between participant-produced estimates and performance by domain.

	Biology	Literature	Math
Prediction versus actual score	0.01	0.12	0.47^***^
Postdiction versus actual score	0.41^**^	0.38^**^	0.35^*^

^*^p <0.05; ^**^p <0.01; ^***^p <0.001 (as compared to 0).

Students were clearly math anxious overall, with an average adapted shortened MARS score of 73/115 (SD = 16.8). Comparatively, Ashcraft and Moore (2009) found an average MARS score of 61/125 using a test instrument with two more questions, across several college samples identified as math anxious. Further analyses suggested that more anxious participants had lower performance and lower levels of overconfidence; however, inferential tests did not reach statistical significance. Therefore we simply conclude that students generally experienced math anxiety. We measured a Cronbach’s alpha of 0.94 for the version of the shortened MARS math anxiety that we used, indicating very good internal consistency. This is comparable to the Cronbach alphas Plake and Parker (1982) found for the original full-length 98-question MARS instrument (α = 0.97) as well as the shortened 24-question version MARS-R (α = 0.98).

Overall, the results were consistent with the unskilled and unaware view, in that there was global overconfidence in predictions across subject domains. The finding that even the students who performed best in math were overconfident (see **Figure 4**) is not consistent with unskilled and unaware, however. Instead of observing underconfidence in the best performers, as has typically been displayed by the unskilled and aware phenomenon, we see persisting overconfidence even in the highest performing participants. In terms of math being unique, it was unique here in the sense that overconfidence was the greatest and metacognitive calibration was the best. Neither of these results would be predicted from the idea that anxiety is particularly high for math.

## General Discussion

Our basic conclusion is that students aligned in some ways to both the unskilled and unaware phenomenon and the idea that math is unique. In both studies, students over-estimated their performance in their predicted scores for all domains, though their calibration did improve for postdictions. This provides support for the domain generality of metacognition under the unskilled and unaware view. Interestingly, the most exaggerated over-confidence was observed for math, which supports the view that math is unique and that metacognition is domain-specific. What is novel about these results is that students appear to be math anxious yet also overconfident in math. In addition, relative calibration of metacognition for math was generally better than other academic subjects. This overconfidence and greater metacognitive calibration replicated in both studies in this paper, and has also been consistent across our earlier studies (Erickson and Heit, 2013). We have reliably documented over past studies that both high school and college students have over-predicted their scores on math tests, along with biology and literature tests.

Math is indeed unique in some respects and this manifests in an interesting way. Rather than showing lower confidence in math compared to other subjects and poorer metacognition, as would be expected based on the original view that math is unique, students generally showed the highest level of overconfidence in math compared other academic subjects. Students, rather than displaying differing metacognition in the form of lower confidence stemming from math anxiety, instead showed differing metacognition in the form of even more exaggerated overconfidence despite the presence of math anxiety. In Study 1, students were retaking only their Algebra 1 class, not a literature or a biology class. Whereas we did not directly measure math anxiety in this study, it is plausible that these students were math anxious overall.

Another relatively novel feature of these two studies is the consideration of both predictions and postdictions of performance, rather than just postdictions or individual test item evaluations. Math was unique in that students displayed higher over-confidence when compared to other subjects, and this persisted in both predictions and postdictions. Students also displayed elements of being unskilled and unaware both before and after the test, though their calibration did improve after taking a test. It seems an obvious result that metacognition would improve after students take a test, and it is tempting to treat postdictions as the more relevant measure to be considered when evaluating actual student metacognition. After all, doing so would help in comparing metacognition across students once they have equal footing in knowing exactly what is on the test. However, we would argue that predictions, not postdictions, provide a more realistic and practical measure of student metacognition. Students typically cannot view actual questions before they take a test in an academic setting. Rather, they must use predictions to guide their self-regulation activities, including studying as well as choices such as how to take notes in class (or even whether to attend class). Students’ metacognitive skill in postdicting performance after a test might be more accurate, but this cannot help them to improve test performance and academic success.

As noted earlier, our findings are focused on the math-anxious student groups studied here, comparing academic subjects. We would see a comparison to non-math-anxious students as a fascinating but challenging potential topic for future research. Just assembling two groups of students who differ in terms of math anxiety but are equal on other variables would present considerable difficulty. Students who differ in math anxiety likely have other attributes (e.g., demographics, math ability) that also differ. These variables would have to be carefully teased apart in order to make any comparisons of math-anxious and non-math-anxious students.

### Possible Mechanisms

It was not the purpose of these studies to find the exact mechanisms that underlie metacognitive function for math. In general, finding a similar pattern of results for two different tasks does not necessarily indicate that they are the same mechanistically. However, theories in the math anxiety literature help explain why metacognition for math is unique when compared to other academic domains. Notably, Beilock (2008) along with others (Ramirez et al., 2013) has shown that working memory is compromised by math anxiety and also by stressful situations, providing a possible explanation for the reduced metacognitive ability we observed for math. Ashcraft and Krause (2007) further showed that math anxiety and peoples’ preoccupation with this fear function as secondary mental tasks that draw on working memory resources necessary for problem solving. Any math problem solving that requires more than simple retrieval of information depends on working memory, so a reduction in working memory capacity can lead to a reduction in math performance. This lower performance then results in a disconnect between typical academic performance and math performance and a corresponding miscalibration in metacognition. People’s metacognitive evaluation of math ability may ordinarily be more accurate when they are engaging in tasks in a less stressful environment but becomes less accurate when put under pressure.

So far, we have demonstrated overconfidence as a flaw in self-monitoring about math and other domains. Self-monitoring strategies such as self-testing give learners specific and measureable feedback about how much they know. Without accurate feedback, learners are unable to select appropriate self-regulation strategies to further their learning process (Dunlosky et al., 2005). Rather, they choose study strategies based on feelings of knowing and judgments of learning, both of which have been shown to be inaccurate self-measures of knowledge (Metcalfe and Finn, 2008). These inaccurate measures lead to student flaws in identifying problem areas of knowledge. Without knowing what they do not understand, learners are unable to make plans to fill in gaps in their knowledge. Students can benefit from using effortful self-monitoring by enacting practice tests at home, where they might not suffer as much detraction from working memory stores. With less stress from a testing environment, they will be able to perform closer to their actual knowledge level. This enables learners to highlight gaps in their knowledge and use this to organize their study time more effectively.

### Possible Limitations

We do not know to what extent these findings generalize to other populations. Both UC Merced and Central Valley High School are both in the same rural area of California and are not necessarily representative of all learners. Ideally, additional studies will be conducted in other learning settings and with more widely differing populations. For instance, populations representing a full spectrum of math anxiety would provide a more complete picture for the relationship between math anxiety and metacognition, though such research would also need to be take account of general anxiety, math ability, and other variables related to math anxiety and metacognition.

Students in Study 2 were math anxious, and although we did not directly measure math anxiety in Study 1, a later study identified another sample of the same high school population as math anxious using the same MARs measure. From this, we might assume that the students in Study 1 had a similar level of math anxiety. Although we did not include a measure of general anxiety, it is also possible that the populations here had general anxiety and not just math anxiety. Presence of generally high anxiety would further compromise working memory and, consequently, metacognitive ability.

Although performance was not exactly the same across academic subjects, it does not appear that the exaggerated overconfidence in math was simply result of choosing intrinsically more difficult test items compared to biology and literature. Questions in both studies were normed for difficulty, and the math test in Study 2 was in fact less difficult than either biology or literature, based on test item difficulty ratings provided by the College Board, creators of the standardized test. Furthermore, analyses that included only those questions that participants performed best on still displayed this finding of exaggerated overconfidence. In an effort to improve the realism of this study, a later version of this study was performed in math classes at UC Merced. Students provided predictions and postdictions for class midterms, thus utilizing a much more realistic assessment than an SAT II test taken in a lab setting. Findings generally replicated those from the studies in this paper, so results in this paper were not an artifact of lab setting or the particular assessments used.

### Final Remarks

We do not doubt that math anxiety exists. However, it is important to differentiate metacognitive judgments of performance from feelings of anxiety, which may have a more emotional or physical, rather than cognitive, basis. That students can be anxious yet overconfident has pernicious implications for struggling math learners. Overconfidence and anxiety provide students with two reasons to avoid studying math or attending math classes. According to models of metacognition, learners stop studying when believe they have reached mastery (Son and Sethi, 2010). Furthermore, extensive evidence shows that anxiety leads to avoidance (Ashcraft, 2002), implying that anxious students would avoid attending math classes. Other examples of math avoidant behaviors include avoiding lectures, avoiding homework, avoiding study time, or avoiding test preparation. Globally, the 2012 Programme for International Student Assessment (PISA) study, which assessed 15–16 year olds in 65 countries, found that students with higher math anxiety were more likely to have lower math self-concept. Trends in this comprehensive study point toward increases in math anxiety over the past decade.

Metacognition can also further impact other abilities such as attention, memory, perception, comprehension, reasoning, and problem solving (Kitchener, 1983; Metcalfe and Shimamura, 1994) and also affect social behavior (Jaccard et al., 2005) and decision making (Cohen et al., 1998). We have not yet examined the effects of math confidence on math anxiety on self-regulation. Which is a better predictor of study behavior, math confidence or math anxiety? Would pointing out the contradiction between being overconfident about math and being anxious about it have beneficial consequences for struggling students? We see these questions as important for future research.

We finish with a cautionary note. Some educational interventions aim to boost students’ math self-confidence, because math self-concept is strongly related to math grades (Marsh et al., 2006). It is important to keep in mind that students’ confidence in their math performance is probably already high, likely contributing to math avoidance. Aiming to further increase self-confidence in math may have unintended consequences for learning.

## Conflict of Interest Statement

The authors declare that the research was conducted in the absence of any commercial or financial relationships that could be construed as a potential conflict of interest.
